# A cold-responsive liquid crystal elastomer provides visual signals for monitoring a critical temperature decrease†

**DOI:** 10.1039/d3mh00271c

**Published:** 2023-04-05

**Authors:** Yuanyuan Zhan, Dirk J. Broer, Junyu Li, Jiuzhi Xue, Danqing Liu

**Affiliations:** a Department of Chemical Engineering and Chemistry, Eindhoven University of Technology Groene Loper 3 5612 AE Eindhoven The Netherlands D.Liu1@tue.nl; b Institute for Complex Molecular Systems (ICMS), Eindhoven University of Technology Groene Loper 3 5612 AE Eindhoven The Netherlands; c Molecular Materials and Nanosystems, Eindhoven University of Technology Groene Loper 3 5612 AE Eindhoven The Netherlands; d Smart Liquid Crystal Technologies Co. Ltd, Jiangsu Industrial Technology Research Institute (JITRI) 280 Huangpujiang Road Chuangshu 215556 China

## Abstract

Critical temperature indicators have been extensively utilized in various fields, ranging from healthcare to food safety. However, the majority of the temperature indicators are designed for upper critical temperature monitoring, indicating when the temperature rises and exceeds a predefined limit, whereas stringently demanded low critical temperature indicators are scarcely developed. Herein, we develop a new material and system that monitor temperature decrease, *e.g.*, from ambient temperature to the freezing point, or even to an ultra-low temperature of −20 °C. For this purpose, we create a dynamic membrane which can open and close during temperature cycles from high temperature to low temperature. This membrane consists of a gold-liquid crystal elastomer (Au-LCE) bilayer structure. Unlike the commonly used thermo-responsive LCEs which actuate upon temperature rise, our LCE is cold-responsive. This means that geometric deformations occur when the environmental temperature decreases. Specifically, upon temperature decrease the LCE creates stresses at the gold interface by uniaxial deformation due to expansion along the molecular director and shrinkage perpendicular to it. At a critical stress, optimized to occur at the desired temperature, the brittle Au top layer fractures, which allows contact between the LCE and material on top of the gold layer. Material transport *via* cracks enables the onset of the visible signal for instance caused by a pH indicator substance. We apply the dynamic Au-LCE membrane for cold-chain applications, indicating the loss of the effectiveness of perishable goods. We anticipate that our newly developed low critical temperature/time indicator will be shortly implemented in supply chains to minimize food and medical product waste.

## Abstract 2

A new generation of critical temperature indicators is developed for monitoring temperature decrease and providing irreversible visual signals. This is realized by using a switchable permeable–impermeable gold-liquid crystal elastomer membrane.

## Graphical abstract

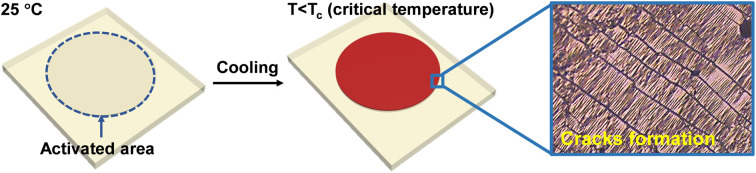

**New concepts**

The aim of this project is to address the issue of storing vaccines, which require a temperature range of 2 °C to 8 °C and may be damaged if the temperature falls below this range. To tackle this problem, we urgently need to develop a new material strategy to monitor temperature decreases. Our project involves the development of critical temperature indicators that provide irreversible visual signals when the temperature drops, such as from 30 °C to −20 °C. We propose a novel design that uses a dynamic membrane consisting of a gold–liquid crystal elastomer (Au-LCE) bilayer structure. This membrane opens up upon temperature reduction, allowing the transport of chemical reagents. In terms of material innovation, we use a cold-responsive LCE, which means that the geometric actuations occur when the temperature decreases, contrary to the conventional thermo-responsive LCEs that respond to temperature increases. To demonstrate the validity of our material concept, we have utilized the newly developed dynamic membrane in a device and exhibited its practical application in a real environment.

## Introduction

Critical temperature indicators (CTIs), which provide consumers with an irreversible visual warning when an object's temperature threshold is surpassed, are emerging technologies being used in monitoring and controlling storage and transporting conditions for perishable products, the quality of which cannot be easily determined by visual inspection alone.^1^ Yet, most of the CTIs on the market today are upper critical temperature indicators showing visual signals when the temperature increases and exceeds the predetermined limit.^2,3^ The existing examples, including the commercial WarmMark, ColdMark, and FreezeSafe time-temperature indicators, are applied in food packaging. However, in the context of vaccines, which are typically stored between 2 °C and 8 °C, are damaged when the environmental temperature exceeds a lower limit.^4^ Therefore, there is an urgent requirement of developing a novel strategy to monitor low critical temperatures next to the existing critical high temperature sensors.

**Fig. 1:**
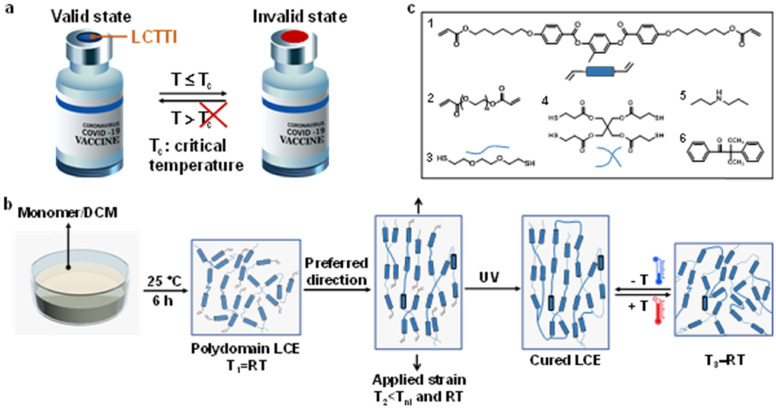
Design of the dynamic membrane. (a) Schematic illustration of the low critical temperature/time indicator (LCTTI) for vaccine temperature monitoring. (b) Fabrication of a cold actuated liquid crystal elastomer (LCE). The monomer mixture was dissolved in DCM and transferred to the mold for the Michael addition reaction at room temperature in the dark. Prior to the photopolymerization, the polydomain elastomer was stretched uniaxially to align the liquid crystal mesogens below nematic–isotropic transition temperature *T*_ni_ and room temperature. Then the nematic monodomains were frozen in upon UV exposure. (c) Chemicals used for the formation of the LCE.

Temperature-responsive materials provide a logical powerful tool for temperature indicators. Thermo-responsive polymers are an advanced class of materials, which convert a thermal signal into a physical, mechanical, optical, or chemical signal and are increasingly utilized in daily life.^5^ The change in optical properties, such as color, is very useful for a warning indication when a critical temperature is exceeded. The most mature and widely developed temperature-responsive polymer is the poly(*N*-isopropylacrylamide) based hydrogel, which can undergo a reversible sol–gel transition at critical temperature.^6–8^ This phase transition is owing to change in hydrophilic and hydrophobic interactions among polymer molecules and water molecules. Nevertheless, this system requires a significant amount of solvent and can only operate in an aqueous environment, which increases the complexity and limits the critical temperature indication range. Contrarily, liquid crystal elastomeric polymer networks (LCEs) are emerging materials currently in development possessing application-specific characteristics, such as artificial muscles for soft robotics.^9–29^ Molecularly aligned LCEs exhibit anisotropic macroscopic deformation typically in response to heat by programming molecular alignment.^30–32^ By temperature change, the LCEs undergo order-to-disorder transformation due to nematic–isotropic phase transition and, as a result, contraction along the molecular director takes place. However, the opposing behavior of macroscopic expansion along molecular alignment, associated with disorder-to-order transition responding to temperature decrease, has been overlooked and barely exploited. The known low temperature LCEs mostly perform at ambient temperature.^33,34^ We further decrease the responsive temperature to the freezing point or even below, based on which we take our materials to the next level by integrating the materials into a device, *viz.* a low critical temperature/time indicator which provides optical indication (Fig. 1a).

In this work, we present a new generation of LCEs that responds to temperature decrease, even towards cold at temperatures as low as −20 °C, with macroscopic anisotropic expansion parallel to the director and shrinkage orthogonal to that. We fabricated our cold-responsive LCE by using a two-step polymerization technique, a primary thiol–acrylate Michael addition reaction followed by a secondary photopolymerization of polymer networks (Fig. 1b). We chose to perform the thiol–acrylate click reaction in a solvent, dichloromethane (DCM), to disrupt the liquid crystal order of the mesogens during the formation of polymer networks as well as to obtain a polymer elastomer with isotropic polydomains at room temperature (RT). The polydomain structure of the LCEs was confirmed by polarized microscopy and X-ray diffraction (XRD) analysis (Fig. S1, ESI†). Upon temperature reduction, the LCE transits from polydomain/isotropic to monodomain uniaxially aligned, causing an expansion along the alignment direction. To regulate the preferred deformation direction, we slightly strain the LCE while maintaining its polydomain state, which is memorized by the photopolymerization below nematic–isotropic transition temperature *T*_ni_ and RT, as determined by DSC (Fig. S2, ESI†). The applied strain was optimized in terms of actuation (Fig. S3, ESI†). Since the photo-crosslinking of the secondary step requires the presence of unreacted acrylates (Fig. 1b), the initial molar ratio of functional group acrylate to thiol was optimized to 1.05 (Table S1, ESI†). Chemicals used for the fabrication of the LCE are shown in Fig. 1c. A commercially available liquid crystal mesogen 1 was used to form the liquid crystal elastomeric network. To bring the response temperature to the critical temperature of commonly used vaccines, *e.g.*, 2 °C to 8 °C, we considered, various methods, such as introducing flexible spacers, using mesogen blends, oligomerization prior to crosslinking and using different crosslinkers.^33,35^ In this paper, we have chosen to introduce flexible backbones by copolymerizing poly(ethylene glycol) diacrylate (PEGDA) derivative 2 with a molecular weight of 300 g mol^−1^. Dithiol monomer 3 and tetra-thiol monomer 4 were chosen as a chain extender and crosslinker, respectively. Catalyst 5 was selected to initiate the thiol–acrylate click reaction. Photoinitiator 6 was used for photopolymerization to freeze in the pre-aligned nematic LCE upon UV illumination. The *T*_ni_ of final cured LCEs is at or below RT, depending on the PEGDA concentration.

**Fig. 2:**
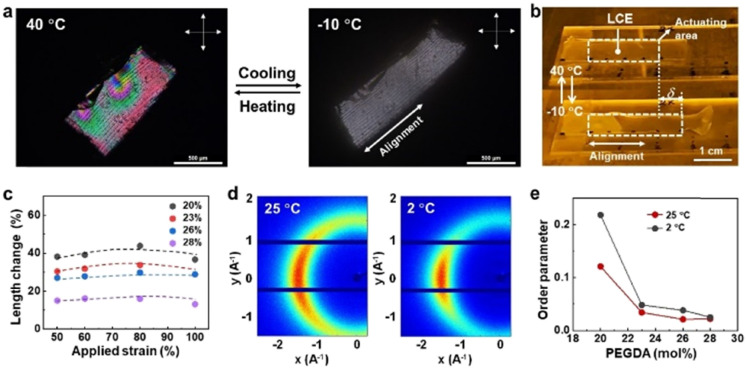
Expansion of LCEs in response to temperature decrease. Elongation of the LCE upon temperature decrease shown in (a) cross polarized optical microscopic images and (b) photograph. The sample with 20 mol% polyethylene glycol (PEGDA) was used. The elongation is with approximately 40% strain (*δ*). (c) Expansion of LCE samples quantified as length change as a function of applied strain prior to photocuring. Dashed lines are to guide the eyes. (d) 2D-XRD images showing the nematic molecular structure of the LCE at 25 °C and 2 °C, respectively. (e) Order parameter, calculated from XRD analysis, of the LCEs at different concentrations of PEGDA.

We observed deformation of the LCEs upon temperature decrease both microscopically and macroscopically by placing them on a coldstage equipped with a precise temperature control monitor. As shown in Fig. 2a and b, upon cooling the LCE uniaxially expands along the pre-determined direction with approximately 40% strain (Videos S1 and S2, ESI†). Since the LCE is weakly crosslinked, slightly above the nematic–isotropic phase transition temperature the liquid crystals are aligned in a polydomain fashion, which changes into the monodomain when reducing the temperature to the nematic phase (−10 °C), as observed between cross polarizers in optical microscopy (Fig. 2a). The expansion strain can be optimized by adjusting the applied strain prior to photocuring. Fig. 2c shows that the threshold strain that allows anisotropic deformation is 50%, below which the LCE deforms isotropically. Above 100% strain, the LCEs fracture (Fig. S3, ESI†). Since the expansion originates from the comparatively disorder-to-order transformation of the molecular structure of liquid crystals, molecular order of the liquid crystal mesogens is expected to increase. This change is confirmed by XRD analysis. Diffraction patterns in Fig. 2d and Fig. S4 (ESI†) exhibit a typical nematic liquid crystal structure regardless of addressed temperature. We calculated the scalar order parameter of the LCEs with various concentrations of PEDGA from XRD data (Fig. S4, ESI†).^36^ We can see from Fig. 2e that the order parameter increases with decreasing temperature. In general, the order parameter is below 0.3 irrespective of temperature and PEGDA concentration. This value is lower than those of typical nematic LCEs,^37^ indicating that the incorporation of isotropic PEGDA molecules disrupts the order of the LCEs.

**Fig. 3:**
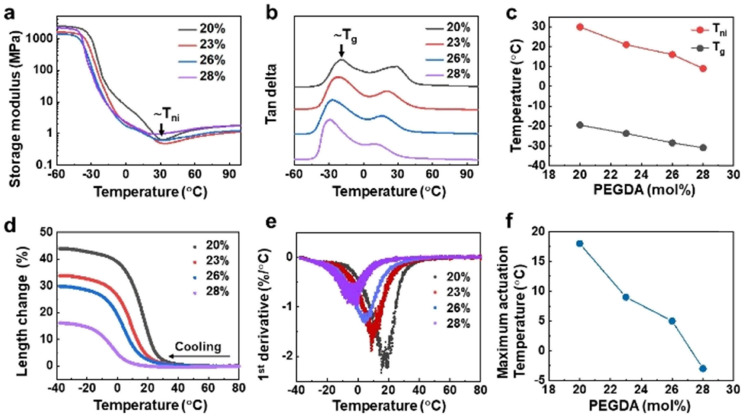
Cold-responsive expansion of LCEs. Dynamic mechanical thermal analysis (DMTA) of the LCEs at various concentrations of PEGDA demonstrating (a) storage modulus and (b) tan delta. (c) Estimated phase transition temperature *T*_i_ and glass transition temperature *T*_g_ of the LCEs as a function of PEGDA concentration. (d) Deformation of the LCE characterized as length change measured by DMTA upon cooling at a rate of 2 °C min^−1^. (e) Actuation rate of the LCE determined by the 1st derivative of the length change. (f) Maximum actuation temperature as collected at the peak of actuation rate as a function of PEGDA concentration.

Next, we investigated the LCEs’ (ultra) low temperature response. We determined the phase transition temperature *T*_ni_ and glass transition temperature *T*_g_ of the LCEs by measuring dynamic modulus and tan delta using dynamic mechanical thermal analysis (DMTA). *T*_ni_ and *T*_g_ are indicated in temperature-resolved storage modulus and tan delta curve, respectively, in Fig. 3a and b. Regardless of PEGDA content, all the *T*_ni_ values are at or below room temperature. Both *T*_ni_ and *T*_g_ decrease linearly when elevating PEDGA concentration (Fig. 3c), as a result of the decrease of mass content of the liquid crystal mesogen in the LCE. Upon further increasing the concentration of PEGDA to 28 mol%, we can even achieve the actuation temperature in the range of −30 °C to 10 °C. Based on this, we examined and quantified the expansion of the LCEs by measuring the length change in the ranges of *T*_ni_ and *T*_g_. We observed from Fig. 3d that all the samples containing different concentrations of PEGDA present length increase upon cooling and the expansion temperature range is consistent with that estimated in Fig. 3c. One can notice that the 20 mol% samples present the maximum length change of 44%, which is larger than those reported in LCEs even of higher actuation temperature in the literature.^38^ When further increasing the PEGDA content, meaning reducing the liquid crystal mesogen content, the strain decreases to minimum 15%. This reduction is owing to the loss of liquid crystal elastomer characteristics. We calculated the actuation rate by performing the first derivative of the length change over temperature. It is estimated that the deformation rate is ranging from 1.0%/°C to 2.3%/°C at such low temperatures, indicating the sharpness in actuation (Fig. 3e). Fig. 3f suggests that the maximum deformation temperature is 10 °C lower than *T*_ni_.

**Fig. 4:**
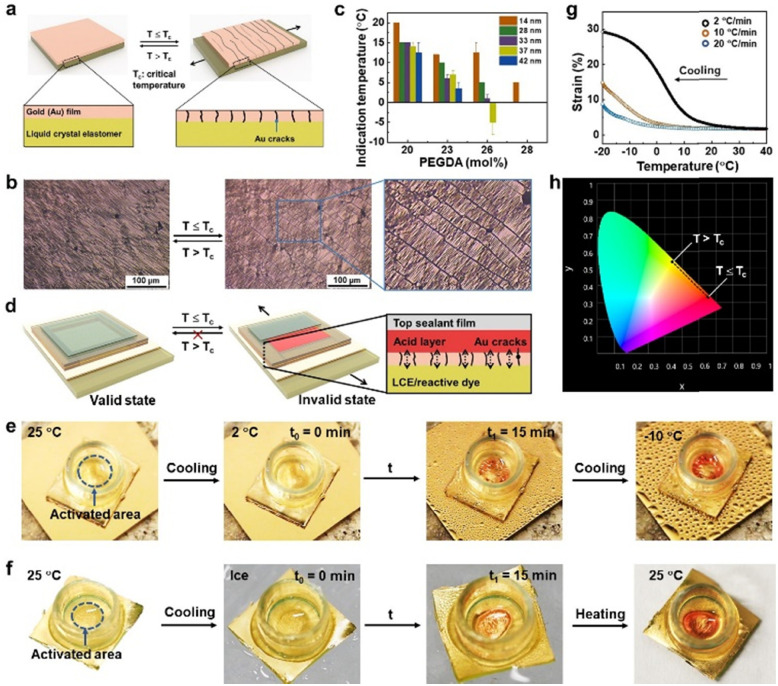
Assembly and demonstration of LCTTIs. (a) Schematic illustration of the working principle of bilayer Au-LCE membrane upon temperature decrease. (b) Optical microscopy images showing the generation of cracks at Au film upon temperature decrease. (c) Comparison of indication temperature among samples containing different concentrations of PEGTA and various thicknesses of Au film. Error bars are obtained from at least three measurements of three specimens of each concentration and Au film thickness. (d) Schematic illustration of the LCTTI construction. (e) Photographs showing the LCTTI demonstrating color changes when cooled from room temperature to 2 °C and −10 °C. (f) Photographs showing the LCTTI demonstrating color changes when placed on an ice surface and staying red when returning to room temperature. The acid container is 4 mm in diameter. (g) Influence of cooling rate on actuation strain. (h) CIE 1931 chromaticity diagram showing the color changes of the device at valid (*T* > *T*_c_) and invalid states (*T* ≤ *T*_c_). In these experiments, the Au film thickness is 33 nm and the PEGDA concentration is 26 mol%.

Our design principle of the low critical temperature/time indicator (LCTTI) is to use the LCE to gate chemical substances, which provides visual signals, to be perceived by eye upon the temperature decrease. In general, the LCE is permeable. To convert it into a switchable permeable–impermeable system on demand, we deposited a metallic gold (Au) top for an Au-LCE bilayer construction. The Au top is firmly adhered to the LCE without any detachment as supported by the thiol chemistry. The thin Au top prevents early appearance of optical signals to avoid false warning. Initially, the Au coating appears slightly wrinkled, as observed by reflective optical microscopy (Fig. 4b and Video S3, ESI†). This is due to mismatch in the thermal expansion coefficients of materials induced stress fields,^39^ independent of long-range orientational order of the LCE. Upon cooling, an in-plane stretching strain parallel to molecular alignment at the Au-LCE interface is exerted on the Au film, which stems from the dimensional change of the LCE. This causes Au to fracture perpendicular to the molecular director (Fig. 4b and Video S3, ESI†). These cracks are the results of a balance between the energy generated by the expanding LCE and the critical shear stress of the relatively stiff Au.^40^ Subsequently, the Au-LCE becomes permeable to chemical substances. In order to create an optical effect, we dispersed a pH-responsive dye, methyl yellow, in the LCE (Fig. S5, ESI†),^41^ which migrates through the Au gates into an aqueous acid environment. This LCTTI construction is shown in Fig. 4d. The protonation of methyl yellow takes place at the Au-LCE interface. As a result, the LCTTI changes from bright yellow into red, the extent of which relies on the number of cracks (Fig. 4e, f and Fig. S6, Video S4, ESI†). After 15 minutes when the rupturing is completed, irrespective of cooling rate (Fig. 4g and Fig. S7, ESI†), the chemical reaction reaches equilibrium and, consequently, the generated color is stabilized. To clearly quantify the displayed color, we employed the CIE 1931 chromaticity diagram (Fig. 4h and Fig. S8, ESI†). Initially, in the valid state, the LCTTI exhibited yellow coordinates at (0.458, 0.541), while in the invalid state the red coordinates at (0.672, 0.328). We can further decrease the onset temperature by increasing either the PEGDA concentration or the Au film thickness (Fig. 4c). We can ascribe this to the less strain per thickness addressed on the film. When returning to the room temperature, the cracks are closed, while the displayed color remains red (Fig. 4f and Fig. S6, ESI†).

## Conclusions

In conclusion, we have developed a new generation of critical temperature indicators that are capable of monitoring temperature decrease and provide irreversible visual signals. This is based on a switchable permeable–impermeable Au-LCE bilayer membrane. Upon temperature decrease, *e.g.*, from 30 °C to −20 °C, the Au-LCE bilayer membrane gates the optical signals and therefore the indicator changes from bright yellow to red. Furthermore, we have effectively regulated the onset temperature in the range of 20 °C to −5 °C by adjusting design parameters such as Au film thickness and LCE composition. We also assessed the durability and stability of the indicators by exposing them to ambient conditions. After six months, no fractures were discovered in the Au film and the reflecting color remained the same. These findings imply that the indicator can be manufactured and stored at room temperature. Therefore, it lowers manufacturing and storage costs, especially if the monitored goods are refrigerated for preservation. With our first demonstrations in simulated cold-chain, we believe that our LCTTI will be extremely valuable to supply chain. To put the scale into context, we envision that our innovative technology can cut global greenhouse gas emissions by reducing perishable product waste.

## Experimental section

### Materials

Liquid crystal mesogen (RM82) was purchased from Merck KGaA. Poly(ethylene glycol) diacrylate (PEGDA-300) and 3,6-dioxa-1,8-octanedithiol (DODT) were purchased from TCI. Pentaerythritol tetrakis(3-mercaptopropionate) (PETMP), dipropylamine (DPA), propionic acid, sulfuric acid, and methyl yellow were obtained from Sigma-Aldrich. Photoinitiator Irgacure 651 was purchased from Ciba. An acid solution of propionic acid and sulfuric acid (0.1 M) with a volume ratio of 90/10 was made and used for the color reaction. All chemicals were used as received without purification.

### LCE fabrication

The LCE was fabricated using a two-step reaction method, Michael addition reaction of weakly crosslinked elastomeric networks followed by photo-polymerization of densely crosslinked elastomeric networks. In the first step, acrylates and thiols were dissolved in dichloromethane (3 mL) for 10 min and DPA (0.5 mol%) was diluted in another 3 mL DCM to avoid localized reaction. Subsequently, 0.5 wt% photoinitiator was added to the solution and homogeneously mixed at room temperature. The mixture was then transferred to a cubic polytetrafluoroethylene mold with a dimension of 4 cm × 4 cm × 1 cm in length, width, and depth, respectively. Addition reaction was performed in the dark at room temperature with an open air atmosphere for 6 h. During the liquid crystal network formation, DCM disrupted the liquid crystal order and isotropic networks with polydomains were formed after the first-step curing. In the second step, prior to exposure to light, the elastomeric networks were mechanically pre-stretched with a certain ratio to align the mesogens and form temporary monodomains. The elongated polymer networks were then polymerized at their nematic phase under UV light illumination using a mercury lamp (Omnicure EXFO S2000) equipped with a liquid nitrogen-affiliated hotstage (Instec).

### Low critical temperature indicator fabrication

The adaptive layer (gold) was deposited onto the LCE using a sputter coater (Q150T Plus, Quorum). Due to the presence of surface patterns of PTFE mold resulting from the turning fabrication method, one side of the LCE is replicated with hill-valley alternated line patterns during oligomerization. The other side exposed to the open air is rather smooth without significant surface patterns, and the adaptive layer was sputtered onto the smooth side to avoid early formation of gold layer defects. The thickness of the gold layer is determined by the sputtering current and time. An acid container made of polymethyl methacrylate (PMMA) with a size of 4 mm in diameter and 3 mm in height was glued on the gold-layer-affiliated LCE side. The PMMA container was made by a laser-cutting technique.

### Characterization

The thickness of the gold layer was measured by using an interferometer (Sensofar). The phase transition temperatures of weakly crosslinked polymer networks and densely crosslinked polymer networks were measured by DSC (Q2000, TA Instruments) at a rate of 10 °C min^−1^. The thermal-induced birefringence change of the LCEs was observed by using a polarized optical microscope (Leica DM2700) equipped with a hotstage (Linkam) at a ramp rate of 5 °C min^−1^. When reaching the desired temperature, the sample was isothermal for 5 min to eliminate thermal lag prior to photographing. The molecular structure of the LCEs was analyzed by X-ray diffraction (XRD) on an instrument from Ganesha Lab equipped with a finely controlled temperature setup. The flight tube and sample holder are all under vacuum in a single housing, with a GeniX-Cu ultra-low divergence X-ray generator. The order parameter was calculated following the equation:^42^
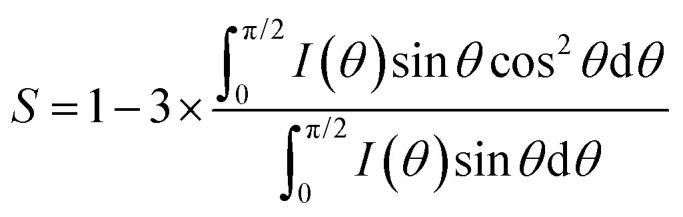
where *θ* is the angle between a molecular symmetry axis and the director.

### DMTA test

Dynamic moduli and phase transition temperatures were recorded by DMTA (Q800, TA Instruments) in frequency–strain mode. Prior to data collection, each sample was thermally cycled between −60 and 100 °C for at least two cycles with a ramp rate of 5 °C min^−1^ to remove thermal history. The test parameters were selected as 0.05 N preload force, 30 μm oscillating amplitude at a frequency of 1 Hz, and 125% force track.

### Actuation measurement

Strain change was measured by DMA in controlled force mode. Before the test, samples were thermally cycled in the range of −40 and 100 °C for three cycles. During the test, the samples were isothermal at 100 °C for 15 min and then cooled down to −40 °C at a rate of 2 °C min^−1^. A bias stress of 3 kPa was applied to determine the length actuation.

## Author contributions

Yuanyuan Zhan (data curation; formal analysis; investigation; methodology; validation; visualization; writing – original draft; and writing – review and editing); Dirk J. Broer (conceptualization; formal analysis; project administration; supervision; and writing – review and editing); Junyu Li (methodology); Jiuzhi Xue (conceptualization); Danqing Liu (conceptualization; funding acquisition; project administration; supervision; and writing – review and editing).

## Conflicts of interest

There are no conflicts to declare.

## Supplementary Material

MH-010-D3MH00271C-s001

MH-010-D3MH00271C-s002

MH-010-D3MH00271C-s003

MH-010-D3MH00271C-s004

MH-010-D3MH00271C-s005
