# Supplementary material for: A cold-responsive liquid crystal elastomer provides visual signals for monitoring a critical temperature decrease
Source: Mater Horiz. 2023 Apr 5;10(7):2649–55. doi: 10.1039/d3mh00271c (PMC10317110; doi:10.1039/d3mh00271c)
Supplement: MH-010-D3MH00271C-s001 [file MH-010-D3MH00271C-s001.pdf]

Electronic Supplementary Material (ESI) for Materials Horizons.
This journal is © The Royal Society of Chemistry 2023

Supplementary Information for

Cold-responsive liquid crystal elastomer provides visual signal for monitoring critical temperature decrease

Yuanyuan Zhan[1,2], Dirk J. Broer[1,2], Junyu Li[3], Jiuzhi Xue[4], Danqing Liu[1,2*]

[1]Department of Chemical Engineering and Chemistry, Eindhoven University of Technology, Groene Loper 3, 5612 AE Eindhoven, The Netherlands

[2]Institute for Complex Molecular Systems (ICMS), Eindhoven University of Technology, Groene Loper 3, 5612 AE Eindhoven, The Netherlands

[3]Molecular Materials and Nanosystems, Eindhoven University of Technology, Groene Loper 3, 5612 AE Eindhoven, The Netherlands

[4]Smart Liquid Crystal Technologies Co. LTD, Jiangsu Industrial Technology Research Institute (JITRI), 280 Huangpujiang Road, Chuangshu, 215556, China

This PDF file includes:

Figures S1 to S8
Captions for Videos S1 to S4

Other Supplementary Information for this manuscript include the following:

Videos S1 to S4

We confirmed the polydomain structure of the LCE by performing XRD analysis. Figure S1 indicates that regardless of PEGDA concentration the LCE exhibits isotropic alignment.

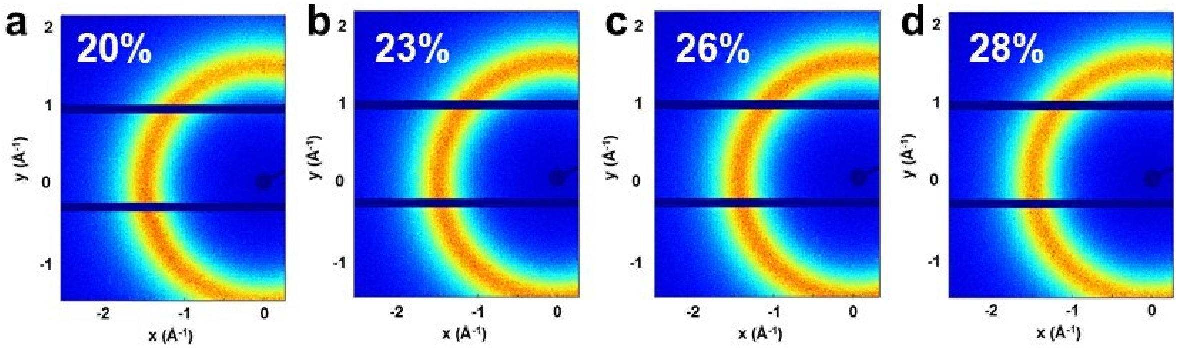

Figure S1. 2D-XRD patterns of the elastomers consisting of different contents of PEGDA ranging from 20 mol% to 28 mol% after first-step thiol-acrylate addition reaction.

The nematic-isotropic transition temperature T_{ni} was determined by DSC measurement. We can see from Figure S2 that regardless of PEGDA concentration T_{ni} is below room temperature. With increasing the PEDTA concentration, T_{ni} slightly decreases.

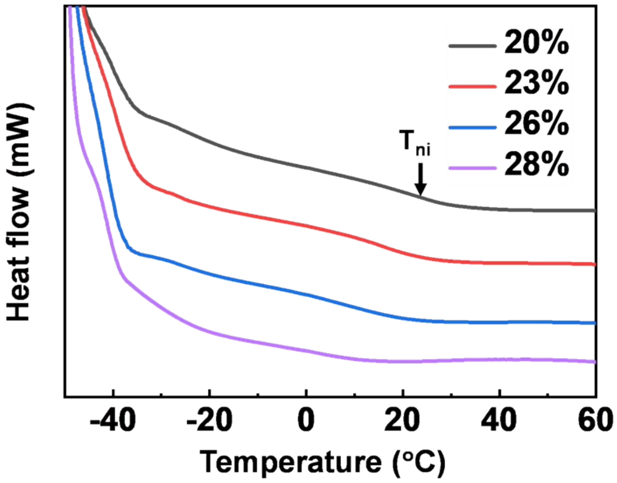

Figure S2. DSC curves showing the transition temperatures of the LCEs consisting of various PEGDA concentration.

We measured the stress-strain curve of the polydomain elastomers after thiol-acrylate addition reaction. Figure S3 shows that the failure strain appears after 100% applied strain, irrespective of PEGDA concentration.

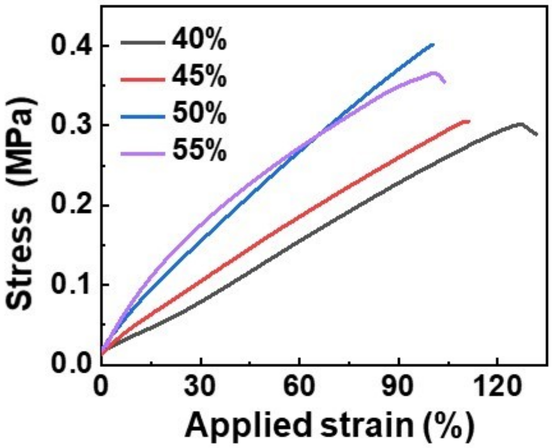

Figure S3. Stress-strain curves of the polydomain elastomers with different PEGDA concentrations after primary thiol-acrylate addition reaction. The experiment was conducted at room temperature.

The compositions of the LCEs are shown in Table S1. The PEGDA concentration out of the total acrylate derivatives is varied from 20 mol% to 28 mol%. The ratio of the rest of composition remains a constant.

Table S1. Compositions for the fabrication of the LCEs.

PEGDA (mol%)	RM82 (mmol)	PEGDA (mmol)	DODT (mmol)	PETMP (mmol)
20%	63	42	92	8
23%	57.75	47.25	92	8
26%	52.5	52.5	92	8
28%	47.25	57.75	92	8

We characterized the molecular structure of the LCE by conducting XRD analysis. The 2D patterns demonstrate the typical nematic liquid crystal structure, regardless of PEGDA concentration and measuring temperature (Figure S4).

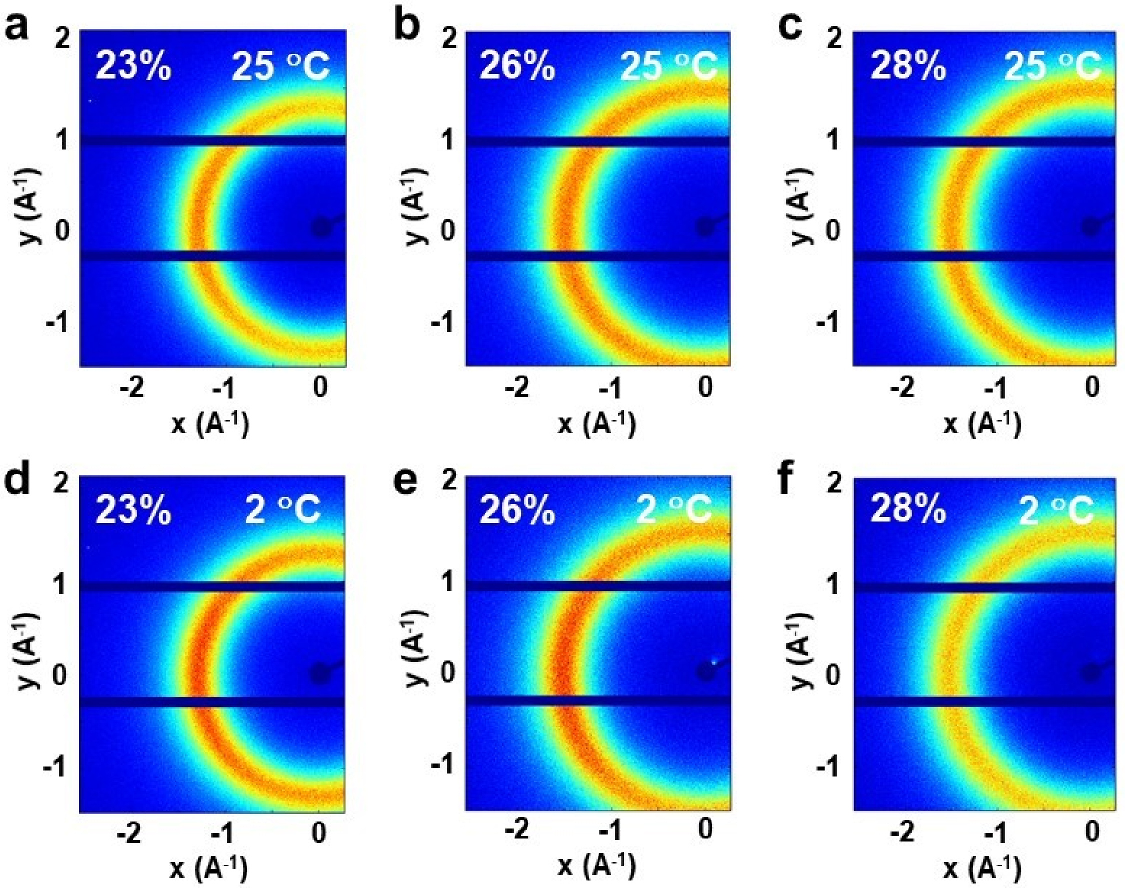

Figure S4. 2D patterns of XRD analysis showing the molecular structure of nematic LCE containing various PEGDA concentrations at room temperature and 2 °C.

We chose methyl yellow as the reactive dye for chemical reaction. The protonation of the dye is shown in Figure S5a. The chemical reaction of methyl yellow dispersed in the LCE was conducted with an acid mixture of propionic acid/sulfuric acid. The color of the LCE changes from yellow to bright red (Figure S5b).

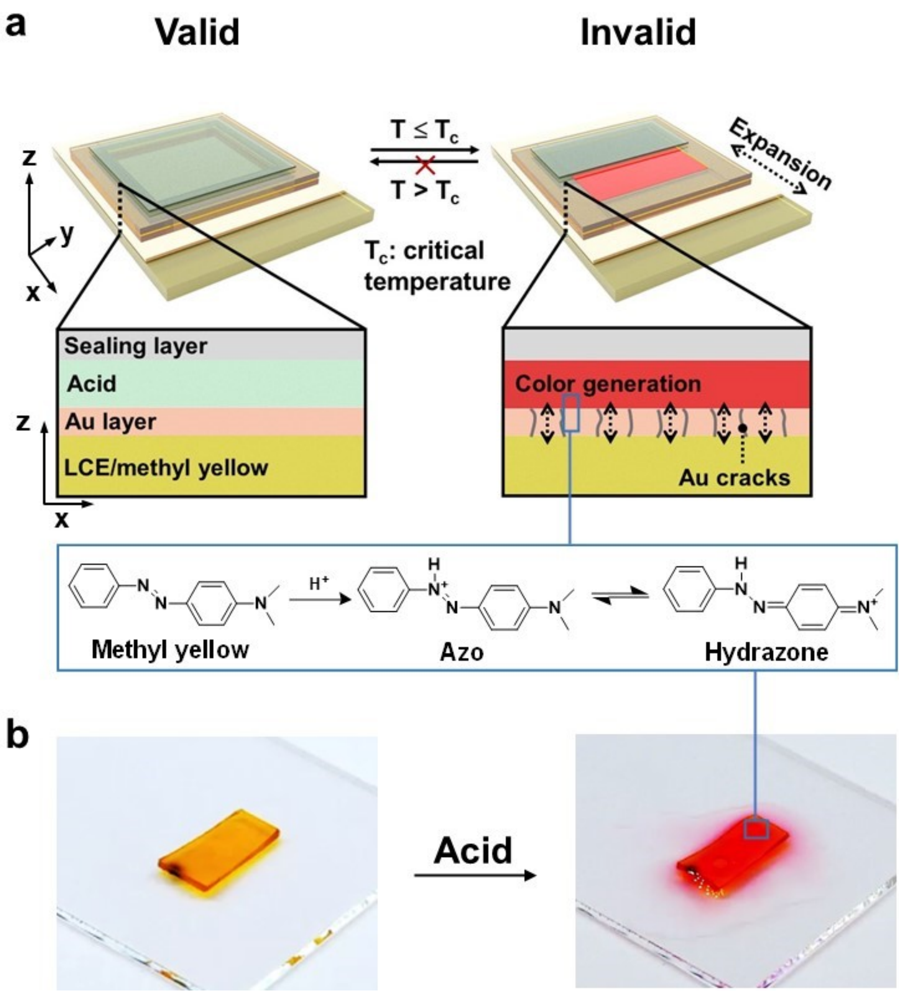

Figure S5. (a) Chemical reaction of methyl yellow. (b) Color change of methyl yellow dispersed in the LCE. The applied acid is a mixture of propionic acid with sulfuric acid (90/10).

The two-dimensional LCTTI is constructed with an Au-LCE, an acid reservoir, and an acid-inert polyethylene sealant film (Figure S6a). As seen from Figure S6b, when jumping from room temperature to freezing point, the color changes from yellow to bright red after 15 min. When returning to room temperature, the color remains red.

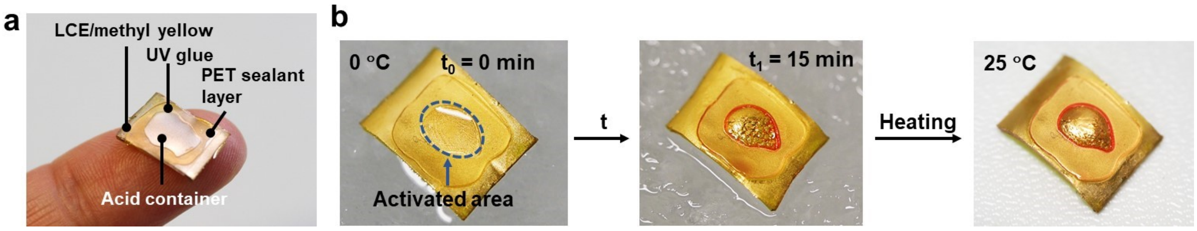

Figure S6. (a) Prototype of two-dimensional (2D) LCTTI. (b) Photographs showing that the 2D LCTTI demonstrating color change when placed on an ice surface and staying red when returning to room temperature. The volume of the applied acid is 30 μL.

We investigated the LCTTI's color exhibition at different temperature decrease rate. When cooling to 2 °C at 20 °C/min, the color appears red in 4 min and stabilizes in 15 min (Figure S7a). In comparison, when jumping from room temperature to 2 °C, the generated red color appears slightly less brighter after 4 min, while the stabilized color after 15 min is comparable (Figure S7b). Based on this, we can conclude that the color exhibition of the LCTTI in 15 min after activation is barely affected by the temperature reduction rate.

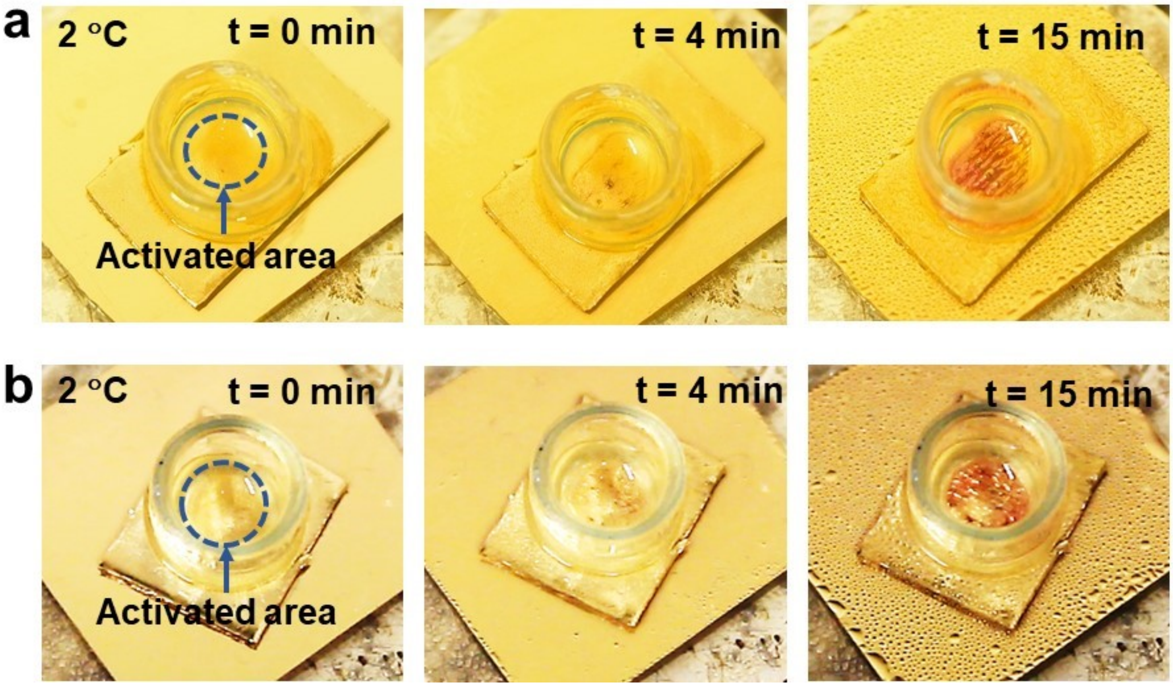

Figure S7. Photographs showing the color change of the LCTTI (a) after cooling down from room temperature to 2 °C at temperature decrease rate of 20 °C/min, and (b) jumping from room temperature to 2 °C.

We characterized the absorbance of methyl yellow by using UV-Vis spectroscopy. The methyl yellow absorbs the light of wavelength ranging from 350 nm to 500 nm and peaks at 410 nm. When treating with an acid, the absorption band shifts to the range of 400 nm to 600 nm with maximum absorption at 510 nm.

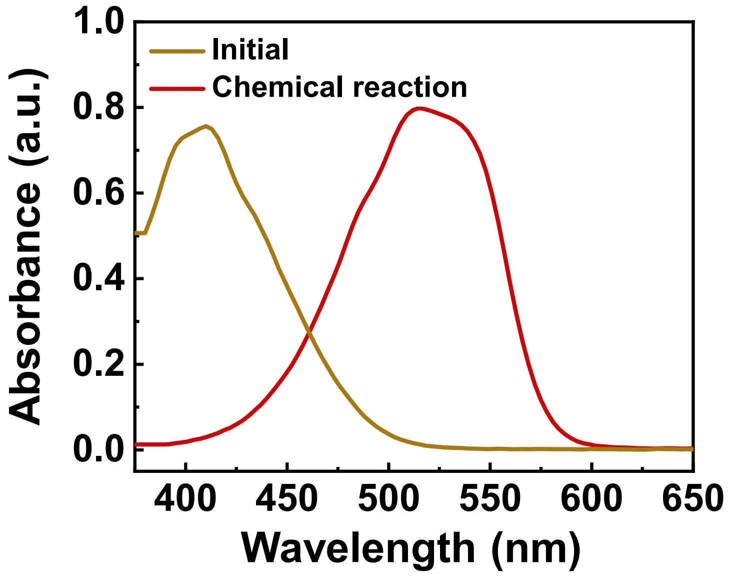

Figure S8. UV-Vis spectra showing the absorbance of methyl yellow before (yellow line) and after (red line) protonation.

Captions for Video S1 to S6

Video S1.
Cross polarized optical microscopy video showing the uniaxial expansion of the LCE upon temperature decrease.

Video S2.

The uniaxial expansion of the LCE induced by temperature decrease recorded by camera.

Video S3.

Optical microscopy video showing the Au film cracking upon temperature decrease.

Video S4.

The LCTTI demonstrating color change when cooled from room temperature to -10 °C.
